# Postoperative Infectious Morbidities of Cesarean Delivery in Human Immunodeficiency Virus-Infected Women

**DOI:** 10.1155/2009/827405

**Published:** 2009-05-25

**Authors:** Helen Cavasin, Thao Dola, Olga Uribe, Manoj Biswas, Mai Do, Azad Bhuiyan, MarkAlain Dery, Chi Dola

**Affiliations:** Tulane Health Sciences Center, Tulane University School of Medicine, LA 70112, USA

## Abstract

*Objective*. To compare the infectious complication rates from cesarean delivery of human immunodeficiency virus (HIV)-infected women and HIV-negative women. *Materials and Methods*. A retrospective analysis was performed on data derived from HIV-infected women and HIV-negative women, who underwent cesarean delivery at two teaching hospitals. Main outcome measures were infectious postoperative morbidity. Descriptive, comparison analysis, and multiple logistic regression analysis were performed. *Results*. One hundred and nineteen HIV-infected women and 264 HIV-negative women delivered by cesarean section and were compared. The HIV-negative women were more likely than the HIV-infected women to deliver by emergent cesarean section (78.0% versus 51.3%, resp., *P* < .05), to labor prior to delivery (69.4% versus 48.3%, resp., *P* < .01), and to have ruptured membranes prior to delivery (63.5% versus 34.8%, resp., *P* < .05). In bivariate analysis, HIV-infected and HIV-negative women had similar rates of post-operative infectious complications (16.8% versus 19.7%, resp., *P* > .05). In a multivariate stepwise logistic analysis, emergent cesarean delivery and chorioamnionitis but not HIV infection were associated with increased rate of post-operative endometritis (odds ratio (OR) 4.10, 95% confidence interval (95% CI) 1.41–11.91, *P* < .01, and OR 3.02, 95% CI 1.13–8.03, *P* < .05, resp.). *Conclusion*. In our facilities, emergent cesarean delivery and chorioamnionitis but not HIV infection were identified as risk factors for post-operative endometritis.

## 1. Introduction

The original landmark study, PACTG 076 reported a decrease in the vertical transmission of human immunodeficiency virus (HIV) infection from mother to child from 25% to 8% with the use of zidovudine in the antenatal, intrapartum, and neonatal periods [1]. More recently, cesarean section has been recommended in women with viral load above 1000 copies/mL to further prevent vertical transmission of HIV [2]. While there are many studies supporting the benefits of the combination of cesarean delivery and antiretroviral medications in preventing vertical transmission of HIV disease from mother to child [3–8], there are also reports on the morbidities that HIV-infected women incurred from surgical procedures [9–11]. Previous reports on the morbidities of cesarean deliveries implied that even healthy, nonimmunodeficient women could sustain significant complications [12, 13]. Thus, it would appear that HIV-infected women may have even more post-operative complications, especially the infectious morbidities, due to their immunodeficient status when they underwent cesarean delivery in an attempt to decrease vertical transmission of the HIV disease.

Current available data reported conflicting results with regard to the post-operative morbidities experienced by HIV-infected women [4, 9–11, 14, 15], therefore, we undertook this study to further examine and compare the infectious complication rate from cesarean delivery of HIV-infected women with those of HIV negative women.

## 2. Materials and Methods

A retrospective analysis was performed on data obtained from women who presented at two teaching medical institutions, the Medical Center of Louisiana at New Orleans - University Campus and Tulane Health Sciences Center, and who subsequently underwent a cesarean section. This study was approved by the Institutional Review Board. Identification of the study patients (HIV-infected women) was obtained by reviewing the labor and delivery log. Their available medical records were reviewed for data abstraction. Control subjects were HIV-seronegative women who delivered by cesarean section during the same time period and whose medical records were available for review.

Medical records were reviewed and data was collected on maternal demographics, number of previous cesarean deliveries, estimated gestational age at time of delivery, classification of cesarean delivery as elective or emergent, pre-operative hematocrit values on the day of delivery, membrane status, application of internal monitoring device, type of anesthesia, skin incision, uterine incision, operative time, estimated blood loss, post-operative morbidities, HIV status, CD4 lymphocyte count, and viral load.

For this study, elective cesarean delivery was defined as a planned operation without signs of labor prior to surgery. Emergent cesarean delivery was defined as surgery performed after the presence of regular uterine contractions with or without rupture of membranes and for maternal and/or fetal indications, such as nonreassuring fetal heart rate status, arrest disorders, and third trimester bleeding.

Post-operative infectious morbidities that were evaluated in this study included endometritis (defined as temperature elevation above 38°C with uterine tenderness and requiring antibiotics treatment in the absence of other etiology for the fever), urinary tract infection, septic pelvic thrombophlebitis, pneumonia, and superficial or deep wound breakdown at the time of discharge from the hospital.

At the above teaching institutions, the management of the patients was conducted by resident physicians under the supervision of Maternal-Fetal Medicine specialist or general obstetrician with consultation from Maternal-Fetal Medicine specialist as indicated. The management of patients was within the standard of current obstetric care. All patients received prophylactic pre-operative antibiotics.

Data was analyzed using SPSS-11 Software (SPSS Inc., Chicago, Ill.). Chi-square analysis with Fischer exact when necessary, Student's *t*-test, and multiple logistic regression analysis were performed when appropriate. Statistical significance was assumed at *P* < .05.

## 3. Results

Over the study period, from July 1998 to December 2004, 119 HIV infected women delivered by cesarean section at the above two teaching institutions. Data was collected on 264 HIV-negative women who delivered by cesarean section during this time and served as the controls. Maternal characteristics of the two groups are presented in Table 1. Overall, the majority of the patients were African American (92.1% of HIV-infected group and 84.3% of HIV-negative group, *P* < .05). Both groups demonstrated a high body mass index (32.7 ± 7.8 kg/m^2^ in HIV-infected group versus 33.6 ± 8.2 kg/m^2^ in HIV-negative group, *P* > .05). The CD_4_ lymphocyte count and viral load near time of delivery of the HIV-infected group were evaluated; 78.8% of the HIV-infected women had a CD_4_ lymphocyte count of ≥200 and 19.2% had undetectable viral load (Table 1).

Obstetrical characteristics of the two groups are compared in Table 2. There was no significant difference between the HIV-infected group and the HIV-negative group in terms of gravidity, parity, number of previous cesarean sections, estimated gestational age at time of delivery, and rate of chorioamnionitis. HIV-infected women were significantly more likely to have a lower preoperative hematocrit than the HIV-negative women (31.9 ± 3.8% versus 33.6 ± 3.8%, resp., *P* < .01). HIV-negative women were significantly more likely than HIV-infected patients to deliver by emergent cesarean section (78.0% versus 51.3%, resp., *P* < .01), to labor prior to delivery (69.4% versus 48.3%, resp., *P* < .01), to have ruptured membranes prior to delivery (63.5% versus 34.8%, resp., *P* < .01), and to have application of internal monitoring devices during labor (31.5% versus 6.0%, resp., *P* < .01).

The surgical characteristics of cesarean delivery in both HIV-infected and HIV-negative women are presented in Table 3. Pfannenstiel skin incision were more often performed in HIV-infected women than their controls (94.5% versus 82.7%, resp., *P* < .01). There was no statistical difference in the other characteristics reviewed which included the type of anesthesia, the type of uterine incision, the estimated blood loss during surgery, or the operative time. Postoperative infectious morbidities are presented in Table 4. There was no significant difference in the infectious morbidities between the HIV infected women and the control group. The most common infectious morbidity after cesarean delivery for our study population is postpartum endometritis.

Furthermore, there was no statistical significant difference between the mean ± SD CD_4_ lymphocyte count between the HIV-infected women with infectious morbidity and those without infectious morbidity (413.2 ± 257.9 cells/mm^3^ and 465.4 ± 283.7 cells/mm^3^, resp., *P* > .05), and between the mean ± SD viral load of those with infectious morbidity and those without infectious morbidity (26,967.1 ± 79,491.6 copies/mL and 43,7242.9 ± 123,389.8 copies/mL, resp., *P* > .05).

Postoperative infectious morbidity in both groups was analyzed according to whether the cesarean section was an elective or emergent delivery. Both groups of women were statistically more likely to experience postpartum endometritis when being delivered by emergent cesarean section than by elective cesarean section, (21.3% versus 3.4%, resp., *P* < .05 in HIV-infected women and 14.6% versus 3.5%, resp., *P* < .05 in HIV-negative women).

Postpartum endometritis composed the majority of the post-operative infectious morbidity, and HIV-negative women in our study had significantly more obstetrics risk factors for postpartum endometritis than the HIV-infected women. Those risk factors were: delivery by emergent cesarean section, rupture of membranes and labor prior to delivery, and insertion of internal monitoring. Because of that, we further performed logistic regression analysis, controlling for these risk factors to determine whether HIV infection play a significant role in postpartum endometritis. To identify risk factors influencing the risk of postpartum endometritis, we constructed a stepwise proportional odds model. HIV infectious status, emergent cesarean delivery, ruptured membranes prior to delivery, application of internal monitoring devices during labor, chorioamnionitis, and preoperative hematocrit were included in the model-building. HIV infectious status, rupture of membranes prior to cesarean delivery, application of internal monitoring devices during labor, and preoperative hematocrit were not found to be significant predictors of endometritis (*P* > .05). However, the odds of having endometritis were almost 4 times higher in emergent cesarean delivery (odds ratio (OR) 4.1, 95% Confidence interval (CI) 1.41–1.91, *P* = .009) and 3 times higher in those with chorioamnionitis during labor (OR 3.02, 95% CI 1.13–8.03, *P* = .027). It should be noted that because colinearity was present between labor prior to delivery by emergent cesarean section and emergent cesarean delivery, we only included emergent cesarean delivery in the multivariate stepwise logistic regression model (Table 5).

## 4. Discussion

According to our study, there was not a significant difference in infectious postoperative morbidity in HIV infected women who delivered by cesarean section when compared to their cohorts of HIV-negative women. However, logistic regression analysis highlighted the increased risk of postpartum endometritis in women who delivered by emergent cesarean section and who had chorioamnionitis during labor, controlling for covariates including HIV infectious status. The HIV infected women in this study were significantly less likely than the control subjects to deliver by emergent cesarean delivery (51.3% versus 78.0%, resp., *P* < .01) and should, therefore, have had a lower rate of postpartum endometritis. Since we observed similar rates of postpartum endometritis in the two groups, despite the fact that HIV-infected women had less risk factors for postpartum endometritis, we can speculate that HIV infection may be a risk factor for postpartum endometritis after cesarean section. However, because of the small sample size, we do not find statistical evidence that HIV infection resulted in higher infectious morbidity after cesarean delivery as reported in some previous studies [8–11, 16–21]. A possible explanation for our result is that we reported on a population of HIV-infected women who were reasonably immunocompetent. The mean ± SD CD_4_ lymphocyte count of our study population was 455.0 ± 277.9 and 78.8% had a CD_4_ lymphocyte count of at least 200 or greater. Most studies reported the increased risk of postoperative infectious morbidities to be associated with the severe degree of immune suppression [9, 17].

Stratified analysis by whether cesarean delivery was emergent or elective in HIV infected women demonstrated that emergent cesarean delivery increased postoperative endometritis over elective cesarean delivery. This is consistent with previous studies which have also demonstrated the highest risk of postoperative morbidity occurring in HIV-infected women who deliver by emergent cesarean section [11, 20, 21]. Therefore as an HIV-infected woman considers elective cesarean delivery or attempt at vaginal delivery, our counseling should include the increased risk of developing postoperative endometritis should her attempt at vaginal delivery fail and emergent cesarean delivery become necessary. We agreed with Marcollet's recommendation that women with a low probability of having a successful vaginal delivery should consider scheduled cesarean delivery [14]. Current ACOG guidelines on recommending elective cesarean delivery for HIV infected women to reduce vertical transmission emphasize the importance of performing the surgery prior to the onset of labor or rupture of membranes [2]. Cesarean delivery performed after the onset of labor or rupture of membranes (i.e., Emergent cesarean delivery) is of unclear benefit with regard to decreasing the vertical transmission risk [2]. Thus, it is important to adhere to the ACOG recommendation on elective cesarean delivery for HIV-infected women as a mean to further reduce vertical transmission rate, as the benefits of cesarean delivery performed after labor or rupture of membranes is unknown and based on current study and other reports [11, 14, 20, 21], maternal infectious morbidity is greater at times of emergent cesarean delivery when compared to elective cesarean delivery.

Limitations of this study include the small number of study patients with severe HIV disease status as determined by CD_4_ lymphocyte counts and viral load quantifications. Thus, we were not able to demonstrate the association between post-operative morbidity among HIV-infected women and the severity of their diseases. Larger study populations are necessary to truly examine the impact of disease status on postoperative morbidity in HIV-infected patients. We also did not evaluate the use of antiretroviral medications and are unable to comment on any possible effects that these medications may have had on postoperative morbidity. This study was also limited due to its retrospective approach to data collection. A large prospective study will better evaluate the post-operative morbidities of cesarean delivery in HIV-infected women.

## Figures and Tables

**Table 1:** Maternal characteristics.

	HIV-infected	HIV-negative	*P*-value
Age (years)	24.6 ± 5.1	25.7 ± 6.7	NS
Race			
* *African American	105 (92.1%)	210 (84.3%)	<.05
* *Others*	9 (7.9%)	39 (15.7%)	<.05
Maternal weight (kg)	88.9 ± 23.1	89.8 ± 23.2	NS
Maternal height (m)	1.6 ± .07	1.6 ± .07	NS
BMI (kg/m^2^)	32.7 ± 7.8	33.6 ± 8.2	NS
Mean CD Count (*n* = 80)	455.0 ± 277.9	—	
Women with CD_4_ count ≥200 cells/mm^3^	63 (78.8%)		
Mean viral load (copies/mL) (*n* = 73)	40,295.8 ± 115,448.8	—	
Women with undetectable viral load (<400 copies/mL)	14 (19.2%)		

Data presented as number and percent or mean ± standard deviation. NS = Not significant. *Others include: Caucasian, Hispanic, and Asian.

**Table 2:** Obstetrical characteristics.

	HIV-infected	HIV-negative	*P*-value
Gravidity	2.9 ± 2.0	2.8 ± 1.9	NS
Parity	1.6 ± 1.8	1.4 ± 1.6	NS
No. of previous c/s			
* *0	64 (58.7%)	146 (56.2%)	NS
* *1	28 (25.7%)	61 (23.5%)	NS
* *2	12 (11.0%)	34 (13.1%)	NS
* *3	3 (2.8%)	13 (5.0%)	NS
* *4	1 (.9%)	6 (2.3%)	NS
* *5	1 (.9%)	0	NS
EGA at delivery (weeks)	37.4 ± 3.1	37.7 ± 3.4	NS
Preoperative Hematocrit (%)	31.9 ± 3.8	33.6 ± 3.8	<.01
Emergent cesarean delivery	61 (51.3%)	206 (78.0%)	<.01
Labor prior to delivery	56 (48.3%)	179 (69.4%)	<.01
Rupture of membranes prior to delivery	40 (34.8%)	165 (63.5%)	<.01
Use of internal monitor	7 (6.0%)	82 (31.5%)	<.01
Chorioamnionitis	5 (4.3%)	18 (6.8%)	NS

Data presented as mean ± standard deviation or number and percent. NS = Not significant.

**Table 3:** Description of surgical procedures.

	HIV-infected	HIV-negative	*P*-value
Anesthesia			
* *General	10 (8.6%)	30 (11.6%)	NS
* *Regional	106 (91.4%)	229 (88.4%)	NS
Skin Incision			
* *Pfannenstiel	104 (94.5%)	215 (82.7%)	<.01
* *Midline	6 (5.5%)	45 (17.3%)	<.01
Uterine Incision			
* *Low transverse	110 (95.7%)	248 (94.7%)	NS
* *Classical/low transverse	5 (4.3%)	14 (5.3%)	NS
* *with vertical extension			
Estimated blood loss (cc)	857.4 ± 311.2	880.0 ± 364.5	NS
Operative time (min)	69.5 ± 34.9	74.9 ± 33.0	NS

Data presented as mean ± standard deviation or number and percent. NS = Not significant.

**Table 4:** Postoperative infectious complications among HIV-infected women and HIV-negative women.

	HIV-infected	HIV-negative	*P*-value
Infectious complications	20 (16.8%)	52 (19.7%)	NS
* *Endometritis	15 (12.6%)	32 (12.1%)	NS
* *Urinary tract infection	2 (1.7%)	15 (5.7%)	NS
* *Superficial wound breakdown	1 (0.8%)	5 (1.9%)	NS
* *Deep wound breakdown	1 (0.8%)	3 (1.1%)	NS
* *SPT*	0	5 (1.9%)	NS
* *Pneumonia	2 (1.7%)	3 (1.1%)	NS

Data presented as number and percent. *SPT = Septic pelvic thrombophlebitis. NS = Not significant.

**Table 5:** Risk factors variables influencing postpartum endometritis in a stepwise odds model.*

Risk factors^†^	Estimate		Odds of postpartum endometritis		*P* value
	*β*	SE^‡^	OR^‡^	95% CI^‡^	
Intercept	−4.62	1.04	—	—	<.0001
Emergent cesarean delivery	1.41	0.54	4.10	1.41–11.91	.009
Chorioamnionitis	1.10	0.50	3.02	1.13–8.03	.027

*Model-building for emergent cesarean delivery: *β*_0_ + *β*_1_ HIV status + *β*_2_ experienced rupture of membranes prior to cesarean delivery + *β*_3_ placement of internal monitoring devices during labor + *β*_4_ Chorioamnionitis + *β*_5_ hematocrit + *β*_6_ indication of cesarean delivery. ^†^Reference group: elective cesarean delivery, absence of chorioamnionitis. ^‡^SE, Standard error; OR, odds ratio; CI, confidence interval.
